# Amyloid pathology and vascular risk are associated with distinct patterns of cerebral white matter hyperintensities: A multicenter study in 3132 memory clinic patients

**DOI:** 10.1002/alz.13765

**Published:** 2024-03-13

**Authors:** J. Matthijs Biesbroek, Mirthe Coenen, Charles DeCarli, Evan M. Fletcher, Pauline M. Maillard, Alzheimer's Disease Neuroimaging Initiative, Frederik Barkhof, Josephine Barnes, Thomas Benke, Christopher P. L. H. Chen, Peter Dal‐Bianco, Anna Dewenter, Marco Duering, Christian Enzinger, Michael Ewers, Lieza G. Exalto, Nicolai Franzmeier, Saima Hilal, Edith Hofer, Huiberdina L. Koek, Andrea B. Maier, Cheryl R. McCreary, Janne M. Papma, Ross W. Paterson, Yolande A. L. Pijnenburg, Anna Rubinski, Reinhold Schmidt, Jonathan M. Schott, Catherine F. Slattery, Eric E. Smith, Carole H. Sudre, Rebecca M. E. Steketee, Charlotte E. Teunissen, Esther van den Berg, Wiesje M. van der Flier, Narayanaswamy Venketasubramanian, Vikram Venkatraghavan, Meike W. Vernooij, Frank J. Wolters, Xu Xin, Hugo J. Kuijf, Geert Jan Biessels

**Affiliations:** ^1^ Department of Neurology and Neurosurgery UMC Utrecht Brain Center Utrecht The Netherlands; ^2^ Department of Neurology Diakonessenhuis Hospital Utrecht The Netherlands; ^3^ Department of Neurology University of California at Davis Davis California USA; ^4^ Department of Radiology & Nuclear Medicine Amsterdam UMC Vrije Universiteit Amsterdam The Netherlands; ^5^ Queen Square Institute of Neurology and Centre for Medical Image Computing University College London London UK; ^6^ Dementia Research Centre UCL Queen Square Institute of Neurology UCL London UK; ^7^ Clinic of Neurology Medical University Innsbruck Innsbruck Austria; ^8^ Department of Pharmacology Yong Loo Lin School of Medicine National University of Singapore Singapore Singapore; ^9^ Memory, Aging and Cognition Center National University Health System Singapore Singapore; ^10^ Department of Neurology Medical University Vienna Vienna Austria; ^11^ Institute for Stroke and Dementia Research (ISD) LMU University Hospital LMU Munich München Germany; ^12^ Medical Image Analysis Center (MIAC) and Department of Biomedical Engineering University of Basel Basel Switzerland; ^13^ Division of General Neurology Department of Neurology Medical University Graz Graz Austria; ^14^ Division of Neuroradiology Interventional and Vascular Radiology Department of Radiology Medical University of Graz Graz Austria; ^15^ Saw Swee Hock School of Public Health National University of Singapore and National University Health System Singapore Singapore; ^16^ Division of Neurogeriatrics Department of Neurology Medical University of Graz Graz Austria; ^17^ Institute for Medical Informatics Statistics and Documentation Medical University of Graz Graz Austria; ^18^ Department of Geriatric Medicine University Medical Center Utrecht Utrecht The Netherlands; ^19^ Department of Medicine National University of Singapore Singapore Singapore; ^20^ Departments of Clinical Neurosciences and Radiology and Hotchkiss Brain Institute University of Calgary Calgary Alberta Canada; ^21^ Alzheimer Center Erasmus MC Erasmus MC University Medical Center Rotterdam The Netherlands; ^22^ Department of Neurology Erasmus MC University Medical Center Rotterdam The Netherlands; ^23^ Department of Internal Medicine Erasmus MC University Medical Center Rotterdam The Netherlands; ^24^ Alzheimer Center Amsterdam Department of Neurology Amsterdam Neuroscience Vrije Universiteit Amsterdam Amsterdam UMC Amsterdam The Netherlands; ^25^ Amsterdam Neuroscience Neurodegeneration Amsterdam The Netherlands; ^26^ MRC Unit for Lifelong Health and Ageing, and the Centre for Medical Image Computing UCL London UK; ^27^ Department of Radiology & Nuclear Medicine Erasmus MC University Medical Center Rotterdam The Netherlands; ^28^ Department of Clinical Chemistry Neurochemistry Laboratory Amsterdam Neuroscience Vrije Universiteit Amsterdam Amsterdam UMC Amsterdam The Netherlands; ^29^ Epidemiology and Data Science Vrije Universiteit Amsterdam Amsterdam UMC location VUmc Amsterdam The Netherlands; ^30^ Raffles Neuroscience Center Raffles Hospital Singapore Singapore; ^31^ Department of Epidemiology Erasmus MC University Medical Center Rotterdam The Netherlands; ^32^ Image Sciences Institute University Medical Center Utrecht Utrecht The Netherlands

**Keywords:** amyloid pathology, arteriolosclerosis, dementia, lesion pattern, white matter hyperintensities

## Abstract

**INTRODUCTION:**

White matter hyperintensities (WMH) are associated with key dementia etiologies, in particular arteriolosclerosis and amyloid pathology. We aimed to identify WMH locations associated with vascular risk or cerebral amyloid‐β_1‐42_ (Aβ42)‐positive status.

**METHODS:**

Individual patient data (*n* = 3,132; mean age 71.5 ± 9 years; 49.3% female) from 11 memory clinic cohorts were harmonized. WMH volumes in 28 regions were related to a vascular risk compound score (VRCS) and Aß42 status (based on cerebrospinal fluid or amyloid positron emission tomography), correcting for age, sex, study site, and total WMH volume.

**RESULTS:**

VRCS was associated with WMH in anterior/superior corona radiata (B = 0.034/0.038, *p* < 0.001), external capsule (B = 0.052, *p* < 0.001), and middle cerebellar peduncle (B = 0.067, *p* < 0.001), and Aß42‐positive status with WMH in posterior thalamic radiation (B = 0.097, *p* < 0.001) and splenium (B = 0.103, *p* < 0.001).

**DISCUSSION:**

Vascular risk factors and Aß42 pathology have distinct signature WMH patterns. This regional vulnerability may incite future studies into how arteriolosclerosis and Aß42 pathology affect the brain's white matter.

**Highlights:**

- Key dementia etiologies may be associated with specific patterns of white matter hyperintensities (WMH).
- We related WMH locations to vascular risk and cerebral Aβ42 status in 11 memory clinic cohorts.
- Aβ42 positive status was associated with posterior WMH in splenium and posterior thalamic radiation.
- Vascular risk was associated with anterior and infratentorial WMH.
- Amyloid pathology and vascular risk have distinct signature WMH patterns.

## 1 BACKGROUND

White matter hyperintensities (WMH) are very common in the elderly and are considered an important hallmark of cerebral small vessel disease (cSVD).^1^ Rather than being a single specific entity, cSVD encompasses arteriolosclerosis, cerebral amyloid angiopathy (CAA), and many other vascular disease mechanisms.^2^, ^3^ In addition, some WMH may potentially be caused by non‐vascular processes including neurodegenerative disorders.^3^, ^4^ In particular amyloid pathology, in the context of CAA or Alzheimer's Disease (AD), is associated with increased WMH burden, some of which may be neurodegenerative and not due to accompanying CAA.^5^, ^6^, ^7^ As such, WMH due to arteriolosclerosis and amyloid pathology are both highly prevalent in the elderly population, and often occur together as mixed pathologies.^8^ It is therefore often challenging to determine to what extent WMH are caused by either of these disease mechanisms in individual patients.

Identifying specific disease mechanisms underlying WMH in order to develop targeted treatments to prevent white matter injury is an important topic. One specific way to improve detection of causes underlying WMH is through detailed analysis of WMH location and patterns. Available evidence suggests WMH in posterior brain regions are associated with cerebral amyloid burden.^5^, ^9^, ^10^ Conversely, arteriolosclerosis‐related WMH may preferentially be located in frontal brain regions.^11^, ^12^, ^13^, ^14^ Two recent examples of using patterns of white matter injury to improved disease classification are the recently updated diagnostic criteria for CAA which now incorporate WMH occurring in a multi‐spot pattern,^15^, ^16^ and a machine learning algorithm for detecting arteriolosclerosis based on regional fractional anisotropy values, WMH volume, and demographics.^17^ However, studies addressing WMH locations associated with specific disease mechanisms are limited in number and sample sizes, have rarely directly compared WMH locations across disease mechanisms, and have sometimes reported conflicting results, possibly explained by methodological differences (statistical approaches and whether or not total WMH volume was accounted for).^18^ Data from large patient samples are therefore needed to establish whether WMH locations associated with either arteriolosclerosis or amyloid pathology are indeed dissociated and to identify white matter locations where WMH are most strongly associated with either of these disease mechanisms.

We aimed to determine which WMH locations are associated with either amyloid‐β_1‐42_ (Aβ42) pathology (based on cerebrospinal fluid [CSF] or positron emission tomography [PET] amyloid biomarkers) or an unfavorable vascular risk profile as an indicator of risk of arteriolosclerosis

## 2 METHODS

### 2.1 Subjects

We selected patients from a recently published Meta VCI Map consortium project^19^ involving 3525 memory clinic patients from 11 cohorts from Austria (1 cohort: PRODEM^20^), Canada (2 cohorts: Brain IMPACT,^21^ FAVR^21^), Germany (1 cohort: VASCAMY), the Netherlands (3 cohorts: ACE, TRACE‐VCI,^22^ UMCC), Singapore (1 cohort: Harmonization^23^), the UK (1 cohort: YOAD^24^), and the USA (2 cohorts: ADNI^25^ [http://adni.Loni.usc.edu; see online supplements for further information], AUCD^26^). The Meta VCI Map consortium aims to perform meta‐analyses on strategic lesion locations for vascular cognitive impairment (VCI) using lesion‐symptom mapping.^27^ The inclusion criteria of this consortium project were (1) the evaluation of patients at an outpatient clinic because of cognitive symptoms; (2) the availability of magnetic resonance imaging (MRI) with T1 and either FLAIR or T2 images; (3) the availability of neuropsychological data. Patients with any degree of symptom severity (ie, subjective cognitive impairment, mild cognitive impairment, dementia) and either presumed vascular, neurodegenerative, or mixed etiology were included. Patients diagnosed with apparent non‐vascular and non‐neurodegenerative causes of cognitive impairment (eg, excessive alcohol consumption, brain tumor, trauma, multiple sclerosis, psychiatric disorder) or monogenic disorders (eg, cerebral autosomal dominant arteriopathy with subcortical infarcts and leukoencephalopathy [CADASIL] or presenilin mutations), were excluded. Cohort‐specific inclusion and exclusion criteria are described in the cited design papers and in the online supplements of the previously published Meta VCI Map consortium project.^19^ Central data processing and analysis were performed at the University Medical Center Utrecht (Utrecht, the Netherlands). An additional inclusion criterion for the current study was the availability of sufficient data on vascular risk factors (see section “vascular risk compound score”) to calculate the vascular risk compound score (VRCS) and/or data on CSF Aß42 levels or amyloid PET imaging, as further specified below.

### 2.2 Cerebral Aß42 status

Aß42 status was assessed with either CSF Aβ42 levels or with amyloid PET imaging. Results were dichotomized (ie, classified as Aβ42‐positive or ‐negative) using local norms in order to enable pooling of multicenter data into a single variable. CSF Aβ42 levels were available in five cohorts. CSF samples in three cohorts from the Netherlands (TRACE‐VCI, UMCC, and ACE) were analyzed in the Neurochemistry laboratory at the Department of Clinical Chemistry of the Vrije Universiteit Amsterdam using Sandwich enzyme‐linked immunosorbent assays (Fujirebio, Ghent, Belgium).^28^, ^29^ CSF Aβ42 levels were dichotomized based on validated cutoff scores of <640 ng/L.^30^ In the YOAD cohort, CSF Aβ42 was analyzed using INNOTEST ELISAs (Fujirebio Europe N.V., Gent, Belgium). Assays were carried out in batches according to local clinical NHNN neuroimmunology laboratory standard operating procedures to achieve a coefficient of variation of <10%. CSF Aβ42 levels were dichotomized at <694 pg/mL. This cut point was determined using data‐driven Gaussian mixture modeling.^31^ Data on CSF Aβ42 levels in the ADNI cohorts were obtained from the ADNI repository (UPENNMSMSABETA2CRM.csv). CSF samples were analyzed by the ADNI Biomarker core laboratory via 2D‐UPLC‐tandem mass spectrometry and adjusted to Aβ42 Certified Reference Material, as described elsewhere.^32^ CSF Aβ42 levels were dichotomized based on validated cutoff scores of <1096 pg/mL.^32^ Amyloid PET imaging was available in one cohort (Harmonization) and the procedure is described elsewhere.^33^ Amyloid positivity on amyloid PET imaging was defined as a standardized uptake value ratio ≥1.5.^33^

**RESEARCH IN CONTEXT**

1. **Systematic review**: The authors reviewed the literature using traditional (eg, PubMed) sources. Amyloid pathology and arteriolosclerosis are key dementia etiologies and are associated with higher white matter hyperintensities (WMH) burden. There are indications from the literature that these etiologies differentially affect posterior (amyloid pathology) versus anterior (arteriolosclerosis) white matter regions.
2. **Interpretation**: We analyzed the relation between WMH location and amyloid‐β_1‐42_ (Aβ42) status and a compound vascular risk compound score (VRCS). The VRCS was primarily associated with WMH in anterior/superior corona radiata, external capsule, and middle cerebellar peduncle, and Aβ42‐positive status with WMH in the splenium of the corpus callosum and posterior thalamic radiation. Thus, amyloid pathology and cardiovascular risk have distinct signature WMH patterns.
3. **Future directions**: Our findings provide novel leads for further research to unravel the mechanisms behind regional vulnerability and resilience to arteriolosclerosis and amyloid pathology, informed by our detailed map of white matter vulnerability to either of these etiologies.

### 2.3 Vascular risk compound score

Data on vascular risk factors included current smoking, hypertension, hypercholesterolemia, diabetes mellitus, obesity (body mass index [BMI] ≥ 30), and history of a vascular event other than stroke were used to calculate the VRCS, similar to two previous studies.^9^, ^34^ The VRCS sums up the number of risk factors for arteriolosclerosis that are present from the total of six aforementioned factors, giving equal weight to each risk factor. To account for missing variables, the compound score is only calculated if data on a minimum of three risk factors are available and expressed as a proportion (ie, the number of present risk factors is divided by the number of available risk factors for each patient). Definitions and harmonization procedures for each of the vascular risk factors are described elsewhere.^19^

### 2.4 Brain MRI processing

MRI protocols and details of the procedures for WMH segmentation and registration are provided in the previously published Meta VCI Map consortium project from which the WMH maps were reused^19^ and only briefly summarized here. Binary WMH segmentations were provided by the participating centers or automatically computed in Utrecht. WMH segmentations were registered to the 1 mm × 1 mm × 1 mm resolution Montreal Neurological Institute (MNI)‐152 brain template for spatial normalization.^35^ All registration results were visually inspected to ensure that the procedure was successful. Failed registrations were excluded (in total 2.7% of patients). Voxels located outside the white matter (defined using the MNI probabilistic white matter atlas,^36^ threshold at 30%) were removed from all individual WMH segmentations to minimize the effect of possible misclassifications of other lesion types as WMH.

The relation between the VRCS and Aβ42 positive status and WMH location was studied at the level of 28 regions of interest (ROIs). ROIs were defined using the ICBM‐DTI‐81 white matter atlas in MNI‐152 space,^37^ which contains 50 ROIs. Bilateral structures were merged into a single ROI, resulting in a reduction of the total number of ROIs to 28. WMH volumes were calculated (in milliliters) and cube root transformed to obtain a normal data distribution prior to performing regression analyses.

### 2.5 Statistical analysis

The VRCS (ranging from 0 to 1) was transformed to a standardized z‐score across cohorts and analyzed as a continuous variable. Aβ42 status was analyzed as a dichotomous variable (ie, Aβ42‐positive or ‐negative). The VRCS and Aβ42 status were analyzed as determinants and regional WMH volumes as dependent variables in separate linear mixed models. All analyses were corrected for age and sex (as fixed effects) and study site (as random effects). Including study site as a random effect accounted for variability in effects across study sites that might arise due to between‐center differences in population or exposure. This approach generally provides more efficient and generalizable estimates compared to including study site as a fixed effect.^38^ All analyses were performed before and after additional correction for total WMH volume. To account for multiple comparisons (ie, to account for 28 separate models for each of the included ROIs), a Bonferroni correction was applied and thus a *p*‐value of <0.002 was considered statistically significant. Reported coefficients (B) are unstandardized. However, the coefficients for the VRCS can be interpreted as standardized coefficients because the independent (VRCS) and dependent variables (WMH volumes) were transformed to z‐scores prior to analysis. The coefficients reported for Aβ42‐positive status are the effect size for Aβ42‐positive versus Aβ42‐negative individuals.

### 2.6 Sensitivity analyses

We performed two sensitivity analyses. First, we repeated the analyses with Aβ42 status as outcome after excluding patients from the TRACE‐VCI cohort. In this cohort, the relation between WMH location and CSF Aβ42 levels had been previously studied, resulting in a significant association between higher Aβ42 burden and higher WMH volumes in the forceps major and posterior thalamic radiation, after correction for age and sex.^9^ This sensitivity analysis served to independently replicate previous findings in a larger dataset by excluding patients from the TRACE‐VCI cohort. Second, all significant associations between higher VRCS or Aβ42 positive status and higher regional WMH volumes were repeated in a smaller dataset (excluding all patients who have missing data on either the VRCS or Aβ42 status) to determine whether differences in WMH locations associated with the VRCS and Aβ42 positive status could be attributed to differences in sample size or characteristics of the patients included in the main analyses.

## 3 RESULTS

Out of the 3525 patients included in the previously published Meta VCI Map consortium project on the relation between WMH location and cognition,^19^ 3132 had sufficient data on either cardiovascular risk factors or Aβ42 status to be included in the current study. The VRCS (mean 0.37, SD 0.31, range 0 to 1) could be calculated in 3117 patients from 10 cohorts. Aβ42 status was available in 1273 patients from six cohorts (based on CSF samples in 1222 patients from ADNI, ACE, TRACE, UMCC, and YOAD, and on amyloid PET in 51 patients from Harmonization), 749 of whom (59%) were Aβ42‐positive. The VRCS and Aβ42 status were not significantly correlated (ANOVA, F = 0.405, *p* = 0.525). Further patient characteristics are provided in Table 1.[Fig alz13765-fig-0001]

**TABLE 1:** Characteristics of included patients.

	Total *n* = 3132
Female, *n* (%)	1544 (49.3)
Age in years, mean (SD)	71.5 (9.0)
Diagnosis, *n* (%)	
SCI	608 (19.4)
MCI	1239 (39.6)
Dementia	1285 (41.0)
CDR, median (IQR)	0.5 (0.5)
Vascular risk factors, *n* (%)	
Current smoking	503 (21.6)^a^
Hypertension	1562 (51.3)^b^
Hypercholesterolemia	1094 (49.0)^c^
Diabetes mellitus	485 (20.4)^d^
Obesity	76 (11.2)^e^
History of vascular event other than stroke or TIA	715 (28.9)^f^
Vascular risk compound score, mean (SD)	0.37 (0.31)^g^
Aβ42 positivity, *n* (%)	749 (59%)^h^
Total normalized WMH volume in milliliter, median (IQR)	6.6 (14.8)

Abbreviations: CDR, Clinical Dementia Rating; IQR, interquartile range; MCI, mild cognitive impairment; SCI, subjective cognitive impairment, TIA, transient ischemic attack; WMH, white matter hyperintensities.

^a^ Missing in 26%.

^b^ Missing in 3%.

^c^ Missing in 29%.

^d^ Missing in 24%.

^e^ Missing in 78%.

^f^ Missing in 21%.

^g^ Available in 3117 patients.

^h^ Available in 1273 patients.

### 3.1 Associations between Aβ42 status and regional WMH volumes

**FIGURE 1 alz13765-fig-0001:**
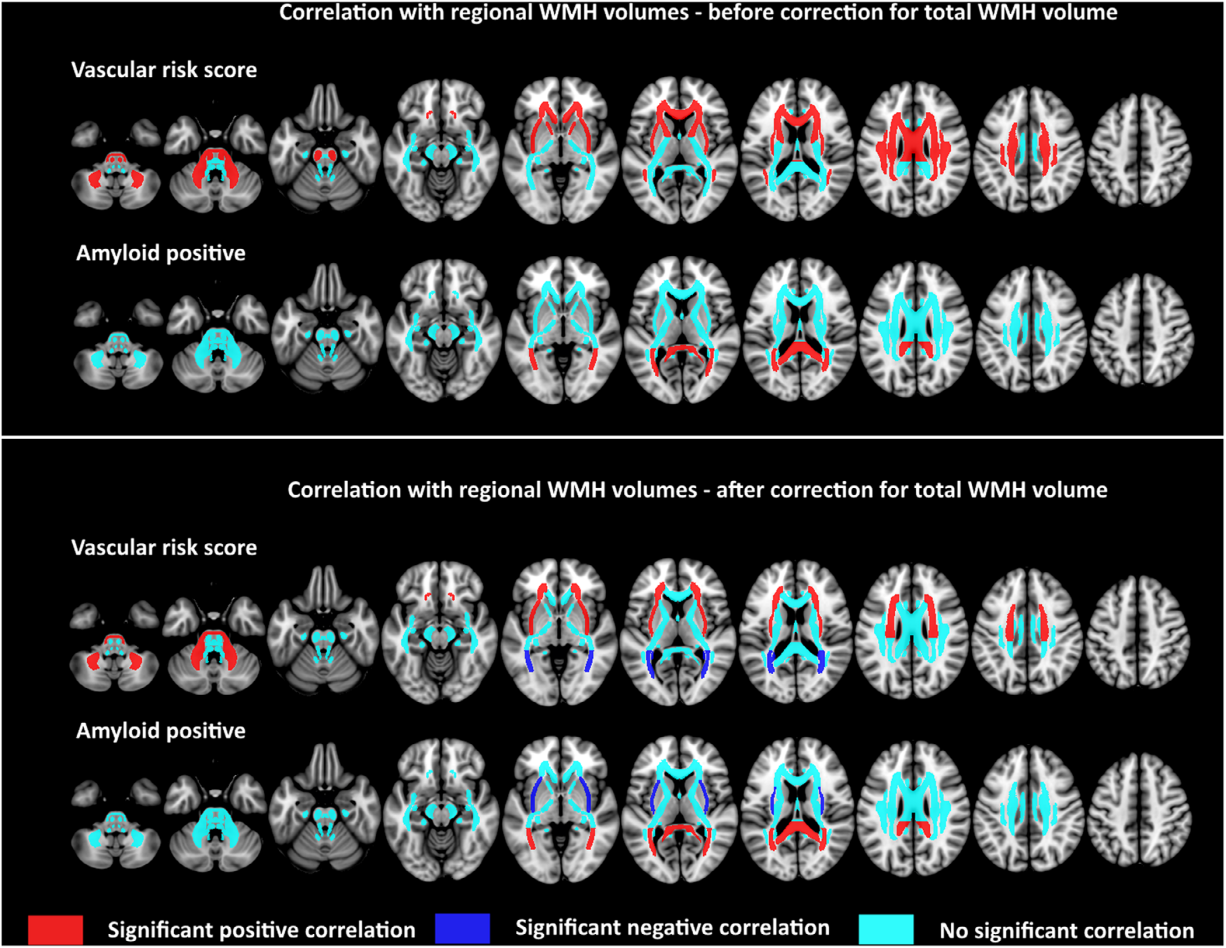
Visual representation of the results of the linear mixed models in which the vascular risk compound score and Aβ42 status were related to regional WMH volumes in 28 ROIs. All analyses were corrected for age, sex, study site, and multiple comparisons. Results before additional correction for total WMH volume are shown in the upper panel and results after additional correction for total WMH volume are shown in the lower panel. Regions with statistically significant positive correlations (indicating either a higher vascular risk compound score or Aβ42 positive status were associated with higher WMH volumes) are shown in red, whereas statistically significant negative correlations are shown in blue. Regression coefficients, standard errors, and *p*‐values for all ROIs are shown in Tables S1 and S2. Aβ, amyloid‐beta; ROI, region of interest; WMH, white matter hyperintensities.

The main analysis concerning the relation between Aβ42‐positive status and regional WMH volumes in 28 ROIs included 1273 patients from six cohorts. After correction for age, sex, and study site, Aβ42‐positive status was associated with higher WMH volume in the splenium of the corpus callosum (B = 0.281, SE = 0.052, *p* < 0.001) and posterior thalamic radiation (B = 0.285, SE = 0.053, *p* < 0.001); no ROIs were associated with Aβ42‐negative status. After additional correction for total WMH volume, the effect sizes attenuated (splenium: B = 0.103, SE = 0.029; posterior thalamic radiation B = 0.097, SE = 0.029), but remained significant (*p* < 0.001), and Aβ42‐positive status was associated with lower WMH volume in the external capsule (B = −0.143, SE = 0.041, *p* < 0.001). The complete list of coefficients, standard errors, and *p*‐values is provided in Table S1 and significant ROIs are shown in Figure 1.

### 3.2 Associations between the VRCS and regional WMH volumes

The main analysis concerning the relation between the VRCS and regional WMH volumes in 28 ROIs included 3132 patients from 11 cohorts. After correction for age, sex, and study site, a higher VRCS was associated with higher WMH volumes in 11 ROIs, namely, the middle cerebellar peduncle (including pons); genu and body of the corpus callosum; corticospinal tract; anterior limb of internal capsule; anterior, superior, and posterior corona radiata; external capsule; superior longitudinal fasciculus; and superior fronto‐occipital fasciculus (B ranging from 0.055 to 0.103; all *p*‐values ≤0.002). No ROIs were associated with lower WMH volume. After additional correction for total WMH volume, a higher VRCS was associated with higher WMH volumes in four ROIs, namely, the middle cerebellar peduncle (B = 0.067, SE = 0.019, *p* < 0.001), anterior (B = 0.034, SE = 0.009, *p* < 0.001) and superior (B = 0.038, SE = 0.008, *p* < 0.001) corona radiata, and external capsule (B = 0.052, SE = 0.014, *p* < 0.001), and a lower WMH volume in two ROIs, namely, the posterior thalamic radiation (B = −0.032, SE = 0.009, *p* < 0.001) and tapetum (B = −0.039, SE = 0.012, *p* < 0.001). Regression coefficients and *p*‐values for all ROIs are provided in Table S2 and significant ROIs are shown in Figure 1.

### 3.3 Sensitivity analyses

In the first sensitivity analysis, excluding patients from the TRACE‐VCI cohort (including 754 patients from the remaining five cohorts with data on Aβ42 status), a significant association was found between Aβ42‐positive status and WMH volumes in the splenium of the corpus callosum (B = 0.290, SE = 0.066, *p* < 0.001) and the posterior thalamic radiation (B = 0.307, SE = 0.069, *p* < 0.001), after correction for age, sex, and study site.

In the second sensitivity analysis, including 1258 patients with available data on both the VRCS and Aβ42 status, the results of the analysis with Aβ42 status as dependent variable were unchanged. In the analyses with the VRCS as dependent variable, the coefficients remained positive for all ROIs and of a similar magnitude for most ROIs. Three out of 11 ROIs remained significant before correction for total WMH volume (ie, anterior and superior corona radiata, and superior fronto‐occipital fasciculus), and one out of four ROIs remained significant after correction for total WMH volume (ie, superior corona radiata). Regression coefficients and *p*‐values for all ROIs are provided in Tables S3 and S4.

## 4 DISCUSSION

In this large multicenter study in patients attending a memory clinic we found that vascular risk factors and Aβ42‐positive status have distinct signature WMH patterns. Vascular risk factors are primarily associated with WMH in the anterior and superior corona radiata, external capsule, and middle cerebellar peduncle, while Aβ42 positive status is associated with WMH in the splenium of the corpus callosum and posterior thalamic radiation.

Prior studies on the relation between WMH location and underlying disease mechanisms have linked vascular risk factors, in particular hypertension,^11^, ^12^, ^13^, ^14^ with anterior WMH, whereas amyloid pathology has been linked with posterior WMH.^9^, ^10^, ^12^, ^13^, ^34^, ^39^ These prior findings have led to the hypothesis that the frontal white matter is more vulnerable to arteriolosclerosis, whereas posterior white matter regions are more susceptible to amyloid pathology and that these pathologies contribute to dementia via regionally distinct pathways.^33^, ^40^ Some studies have suggested that anterior WMH are also linked with amyloid pathology.^41^, ^42^ This discrepancy could be explained by modest sample sizes, relatively crude visual WMH grading methods, and perhaps most importantly, by not correcting for total WMH volume. Both cardiovascular risk factors and AD are associated with higher total WMH volume compared to controls,^12^, ^43^ and the probability that specific white matter regions are affected by WMH increases with higher total WMH volume, which may confound associations between WMH location and etiology. In the current study, including the largest sample size to date, we established that associations between WMH and either risk of arteriolosclerosis or Aβ42 pathology indeed follow the previously suggested anterior‐posterior dissociation and now provide a fine‐grained anatomical map of specific white matter regions where WMH are linked with risk of arteriolosclerosis (external capsule, anterior and superior corona radiata, and middle cerebellar peduncle including the pons) or Aβ42 pathology (posterior thalamic radiation and splenium of corpus callosum). Notably, the middle cerebellar peduncle (which includes part of the pons) had not been included in most prior studies and may therefore have been overlooked as a preferential WMH location in patients at risk of arteriolosclerosis by these studies. This finding fits prior observations from a histopathological case‐control study,^44^ in which pontine WMH (which the authors called pontine ischemic rarefaction) corresponded with arteriolosclerosis on histopathological post‐mortem examination in two patients, and in vivo MRI studies in patients with sporadic atherosclerosis^45^ and CADASIL.^46^ Furthermore, by comparing results before and after correction for total WMH volume, we found that after total volume correction, many regions were no longer significantly associated with either the VRCS or Aβ42 pathology, indicating that these associations may have been mediated by total WMH volume, and that several associations remained significant providing strong evidence that WMH in these regions are linked with either arteriolosclerosis or Aβ42 pathology. Furthermore, after correcting for total WMH volume, the VRCS and Aβ42 positive status were associated with lower WMH volumes in several regions, which is a novel observation that may suggest that these regions are relatively resilient with respect to arteriolosclerosis or Aβ42 pathology. Of note, the sample size in the main analysis was larger for the VRCS (*n* = 3132) than for Aβ42 status (*n* = 1273) positivity, which may affect statistical power. We therefore performed a sensitivity analysis restricted to patients who had data available for both the VRCS and Aβ42 status (*n* = 1258). In this analysis, which confirmed the main finding that Aβ42‐positive status is associated with posterior WMH and higher CRVS with more anterior WMH, although the number of ROIs with a statistically significant association with the VRCS was lower.

The regional vulnerability of the white matter to either arteriolosclerosis or amyloid pathology may have important implications for our understanding of the mechanisms involved in white matter injury. Future research into the mechanism behind regional vulnerability to specific types of injury might ultimately provide novel diagnostic biomarkers and treatment targets. Several possible explanations for the link between WMH location and arteriolosclerosis and amyloid pathology have been proposed in the literature. It has been suggested that arteriolosclerosis (due to hypertension and other cardiovascular risk factors) mainly affects the frontal penetrating arterioles resulting in ischemic injury and anterior WMH.^40^, ^47^ However, to our knowledge, it remains unclear why posterior circulation arterioles are more resilient to the effects of arteriolosclerosis. In contrast, WMH in the context of amyloid pathology may result from several mechanisms. The most straightforward and established mechanism is ischemic injury due to amyloid deposition and dysfunction of the small vessels (ie, CAA), which preferentially affects vessels in posterior brain regions.^48^ Even though vascular amyloid depositions in CAA are mainly located in leptomeningeal and cortical vessels^49^ and are sparse in vessels in the white matter,^50^ CAA is associated with a higher burden of WMH.^51^ However, there is a possibility that the association of posterior WMH with Aβ42‐positive status might also reflect other mechanisms than CAA, based on several observations. First, a recent study found that the association between posterior WMH and amyloid persisted after correcting for cortical microbleeds (which are the main imaging feature of CAA), although it might be countered that microbleeds occur at a later stage of the disease and do not fully capture early changes due to CAA.^9^ Second, a predominance of posterior WMH has been demonstrated in patients with monogenetic AD (ie, in absence of CAA).^34^ Third, a recent study showed distinct patterns of WMH when contrasting CAA (ie, subcortical WMH) with AD (parietal WMH).^14^ Along this line, it has been suggested that non‐vascular pathways for amyloid‐related white matter injury exist, involving either a direct local effect of amyloid deposition in the brain white matter or Wallerian degeneration due to cortical neuronal cell death in the context of AD.^4^, ^52^ In the current study, we cannot discriminate between vascular and parenchymal amyloid given that amyloid biomarkers in CSF do not reliably discriminate between CAA and AD. It has been suggested that Aβ42 status is mainly a biomarker for AD and Aβ40 for CAA, but a recent meta‐analysis found CSF Aβ42 levels to be equally lowered in CAA and sporadic AD.^53^ Another important consideration is that the question of causality concerning the relation between amyloid and WMH has not been unequivocally resolved. Possible mechanistic interactions between WMH and amyloid include the following: (1) WMH may occur as a consequence of amyloid‐related injury as discussed above; (2) WMH and amyloid may be spatially linked as a result of a shared underlying disease mechanisms, for example, blood–brain barrier (BBB) dysfunction resulting in BBB leakage, diminished amyloid‐ß clearance, inflammation, and synaptic and neuronal injury, ultimately leading to both WMH and Alzheimer pathology;^54^ and (3) ischemic injury and WMH may even precede or precipitate amyloid accumulation, as some studies have suggested.^5^, ^42^, ^54^ The results of the current study provide a novel angle for future studies to address these important questions, by studying arteriolosclerosis, Aβ42 deposition, and WMH in the regions that we found to be either predominantly affected or spared in these pathologies, ideally using a longitudinal study design. Furthermore, lesion‐network mapping studies might be useful to determine whether WMH locations associated with Aß42 pathology can be mapped to specific brain networks, given the possibility that Aß42 pathology might involve prion‐like disease propagation through connected brain regions.^55^, ^56^ Another implication of signature WMH patterns associated with arteriolosclerosis and Aβ42‐positive status is that an in‐depth analysis of WMH patterns might have potential as a diagnostic biomarker. A quantitative WMH location‐based score or a machine learning algorithm might be able to identify disease‐specific WMH patterns and estimate the probability that WMH in an individual patient are related to arteriolosclerosis, Aβ42 pathology, or other causes.

The main strengths of the current study are the large sample size, multicenter design, rigorous control for multiple comparisons, and the relatively high spatial resolution achieved by analyzing 28 distinct white matter regions in both the supratentorial and infratentorial white matter. We externally validated our prior finding in the TRACE‐VCI cohort that WMH in the posterior thalamic radiation and forceps minor are associated with Aβ42 status, which further strengthens this conclusion. Limitations are the cross‐sectional study design which hampers causal inferences concerning the observed link between WMH in specific regions and either Aβ42 pathology or the VRCS. The multicenter study design resulted in heterogeneity concerning definitions for vascular risk factors and Aβ42‐positive status (corresponding with locally established cut values) and MRI protocols, and cohort‐specific inclusion criteria may affect the generalizability to the general memory clinic population. Amyloid status was evaluated with either CSF (*n* = 1222), or amyloid PET (*n* = 51). There is evidence that both measures are equally accurate in identifying patients with amyloid pathology in the context of AD.^57^ Moreover, all statistical analyses in the current study were corrected for study site, minimizing the risk of cohort differences due to methodological variations across cohorts. Furthermore, whereas the CSF and amyloid PET biomarkers that were used to define amyloid status have been validated and incorporated in diagnostic criteria for AD,^58^ the VRCS that we used as proxy for arteriolosclerosis of the cerebral small vessels has not been validated for this purpose. Brain autopsy remains the gold standard for diagnosing arteriolosclerosis of cerebral small vessels. Arteriolosclerosis is strongly associated with cardiovascular risk factors, in particular hypertension.^59^ However, we cannot rule out the possibility that the WMH locations associated with the VRCS reflect other vascular (or non‐vascular) disease mechanisms than arteriolosclerosis. Achieving the large sample sizes required for the analyses that we performed in the current study would be challenging if brain autopsy is used to diagnose arteriolosclerosis.

In conclusion, arteriolosclerosis and Aβ42 pathology have distinct signature WMH patterns. In this study, we provide the most comprehensive overview of WMH locations associated with either of these disease processes thus far. The findings provide leads for further research to unravel the mechanisms behind regional vulnerability and resilience to arteriolosclerosis and amyloid pathology.

## CONFLICT OF INTEREST STATEMENT

F.B. is supported by the NIHR biomedical research center at UCLH and reports the following: steering committee or Data Safety Monitoring Board member for Biogen, Merck, ATRI/ACTC, and Prothena; consultant for Roche, Celltrion, Rewind Therapeutics, Merck, IXICO, Jansen, and Combinostics; research agreements with Merck, Biogen, GE Healthcare, and Roche; co‐founder and shareholder of Queen Square Analytics Ltd. M.D. received honoraria for lectures from Bayer Vital and Sanofi Genzyme and reports the following: consultant for Hovid Berhad and Roche Pharma, member of scientific advisory board for Biogen. R.W.P. received honoraria from GE Healthcare and is co‐lead of Neurofilament light consortium. C.H.S. is supported by an Alzheimer's Society Fellowship and is a scientific advisor to BrainKey. V.V. is supported by JPND‐funded E‐DADS project (ZonMw project #733051106). The remaining authors have nothing to disclose. Author disclosures are available in the Supporting Information.

## CONSENT STATEMENT

For all cohorts, ethical and institutional approval were obtained as required by local regulations, including informed consent, to allow data acquisition and data sharing.

## Supporting information

Supporting Information

Supporting Information
