# Supplementary material for: Amyloid pathology and vascular risk are associated with distinct patterns of cerebral white matter hyperintensities: A multicenter study in 3132 memory clinic patients
Source: Alzheimers Dement. 2024 Mar 13;20(4):2980–9. doi: 10.1002/alz.13765 (PMC11032573; doi:10.1002/alz.13765)
Supplement: Supplementary file 1 — Supporting Information [file ALZ-20-2980-s001.docx]

**Supplementary data**

**Supplementary methods**

Alzheimer’s Disease Neuroimaging Initiative

Data used in the preparation of this article were obtained from the Alzheimer’s Disease Neuroimaging Initiative (ADNI) database (adni.loni.usc.edu). The ADNI was launched in 2003 as a public-private partnership, led by Principal Investigator Michael W. Weiner, MD. The primary goal of ADNI has been to test whether serial magnetic resonance imaging (MRI), positron emission tomography (PET), other biological markers, and clinical and neuropsychological assessment can be combined to measure the progression of mild cognitive impairment (MCI) and early Alzheimer’s disease (AD).

**Abbreviations**

MCP: middle cerebellar peduncle. PCT: pontine crossing tract. GCC: genu corpus callosum. BCC: body corpus callosum. SCC: splenium corpus callosum. FX: fornix body and column. CST: corticospinal tract. ML: medial lemniscus. ICP: inferior cerebellar peduncle. SCP: superior cerebellar peduncle. CP: cerebral peduncle. ALIC: anterior limb of the internal capsule. PLIC: posterior limb of the internal capsule. RLIC: retrolenticular part of the internal capsule. ACR: anterior corona radiata. SCR: superior corona radiata. PCR: posterior corona radiata. PTR: posterior thalamic radiation. SS: sagittal stratum. EC: external capsule. CGC: cingulum in the cingulate cortex. CGH: cingulum in the hippocampus. FXST: fornix and stria terminalis. SLF: superior longitudinal fasciculus. SFO: Superior fronto-occipital fasciculus. IFO: Inferior fronto-occipital fasciculus. UNC: uncinate fasciculus. TAP: tapetum.

**Supplementary Table 1. Association between regional WMH volumes and amyloid status.**

| Region | Model 1: corrected for age, sex, study site | | | Model 2: additionally corrected for total WMH volume | | |
| --- | --- | --- | --- | --- | --- | --- |
|  | B | SE | p-value | B | SE | p-value |
| Total volume | 0.218 | 0.051 | **2.7x10-5*** | NA | NA | NA |
| MCP | -0.071 | 0.058 | 0.215 | -0.099 | 0.057 | 0.080 |
| PCT | -0.094 | 0.058 | 0.101 | -0.115 | 0.058 | 0.044 |
| GCC | 0.109 | 0.053 | 0.042 | -0.049 | 0.038 | 0.199 |
| BCC | 0.144 | 0.053 | 0.007 | -0.042 | 0.030 | 0.165 |
| SCC | 0.281 | 0.052 | 6.4x10-8* | 0.103 | 0.029 | 0.0004* |
| FX | 0.097 | 0.056 | 0.080 | 0.080 | 0.056 | 0.151 |
| CST | -0.107 | 0.057 | 0.062 | -0.136 | 0.057 | 0.017 |
| ML | -0.082 | 0.057 | 0.144 | -0.094 | 0.057 | 0.097 |
| ICP | -0.128 | 0.058 | 0.026 | -0.144 | 0.058 | 0.010 |
| SCP | 0.029 | 0.059 | 0.582 | 0.038 | 0.059 | 0.485 |
| CP | 0.023 | 0.058 | 0.701 | 0.022 | 0.058 | 0.726 |
| ALIC | 0.060 | 0.051 | 0.250 | -0.091 | 0.038 | 0.016 |
| PLIC | -0.050 | 0.055 | 0.373 | -0.141 | 0.052 | 0.006 |
| RLIC | 0.020 | 0.050 | 0.691 | -0.111 | 0.040 | 0.006 |
| ACR | 0.152 | 0.053 | 0.004 | -0.032 | 0.030 | 0.282 |
| SCR | 0.122 | 0.053 | 0.022 | -0.071 | 0.027 | 0.009 |
| PCR | 0.138 | 0.053 | 0.009 | -0.064 | 0.026 | 0.013 |
| PTR | 0.285 | 0.053 | 8.7x10-8* | 0.097 | 0.029 | 0.0007* |
| SS | 0.138 | 0.050 | 0.006 | 0.003 | 0.041 | 0.932 |
| EC | -0.003 | 0.052 | 0.938 | -0.143 | 0.041 | 0.0005* |
| CGC | 0.090 | 0.056 | 0.113 | 0.046 | 0.056 | 0.413 |
| CGH | 0.020 | 0.059 | 0.689 | 0.023 | 0.059 | 0.649 |
| FXST | 0.023 | 0.035 | 0.512 | -0.003 | 0.035 | 0.939 |
| SLF | 0.084 | 0.049 | 0.086 | -0.081 | 0.030 | 0.006 |
| SFO | 0.124 | 0.052 | 0.019 | -0.029 | 0.039 | 0.451 |
| IFO | 0.084 | 0.054 | 0.115 | -0.027 | 0.047 | 0.576 |
| UNC | 0.093 | 0.058 | 0.100 | 0.088 | 0.058 | 0.135 |
| TAP | 0.132 | 0.050 | 0.008 | -0.024 | 0.034 | 0.463 |

28 ROIs were analyzed in 1273 patients from 6 cohorts using linear mixed models. Study site was included as random effect and WMH volumes, age, sex and amyloid status (positive or negative) as fixed effects. WMH volumes were cube root transformed. WMH volumes were standardized (i.e. converted to z-scores). *Statistically significant after Bonferroni correction for multiple comparisons.

**Supplementary Table 2. Association between regional WMH volumes and vascular risk compound score.**

| Region | Model 1: corrected for age, sex, study site | | | Model 2: additionally corrected for total WMH volume | | |
| --- | --- | --- | --- | --- | --- | --- |
|  | B | SE | p-value | B | SE | p-value |
| Total | 0.071 | 0.017 | 4.0x10-5* | NA | NA | NA |
| MCP | 0.082 | 0.019 | 2.9x10-5* | 0.067 | 0.019 | 0.0005* |
| PCT | 0.046 | 0.019 | 0.015 | 0.034 | 0.019 | 0.071 |
| GCC | 0.074 | 0.017 | 2.8x10-5* | 0.021 | 0.011 | 0.059 |
| BCC | 0.076 | 0.018 | 1.7x10-5* | 0.014 | 0.009 | 0.121 |
| SCC | 0.037 | 0.017 | 0.034 | -0.024 | 0.009 | 0.011 |
| FX | -0.024 | 0.018 | 0.177 | -0.030 | 0.018 | 0.090 |
| CST | 0.062 | 0.019 | 0.001* | 0.049 | 0.019 | 0.010 |
| ML | 0.032 | 0.019 | 0.103 | 0.021 | 0.019 | 0.274 |
| ICP | 0.023 | 0.019 | 0.249 | 0.019 | 0.019 | 0.034 |
| SCP | 0.027 | 0.020 | 0.183 | 0.025 | 0.020 | 0.212 |
| CP | 0.025 | 0.019 | 0.203 | 0.018 | 0.019 | 0.367 |
| ALIC | 0.066 | 0.016 | 2.5x10-5* | 0.020 | 0.011 | 0.064 |
| PLIC | 0.052 | 0.018 | 0.005 | 0.018 | 0.017 | 0.277 |
| RLIC | 0.027 | 0.017 | 0.123 | -0.023 | 0.013 | 0.076 |
| ACR | 0.094 | 0.017 | 4.1x10-8* | 0.034 | 0.009 | 0.0001* |
| SCR | 0.101 | 0.017 | 3.9x10-9* | 0.038 | 0.008 | 1.4x10-6* |
| PCR | 0.055 | 0.018 | 0.002* | -0.008 | 0.008 | 0.313 |
| PTR | 0.029 | 0.018 | 0.097 | -0.032 | 0.009 | 0.0005* |
| SS | 0.024 | 0.018 | 0.184 | -0.025 | 0.013 | 0.062 |
| EC | 0.103 | 0.018 | 1.0x10-8* | 0.052 | 0.014 | 0.0001* |
| CGC | 0.027 | 0.019 | 0.165 | 0.001 | 0.018 | 0.989 |
| CGH | -0.012 | 0.019 | 0.481 | -0.018 | 0.018 | 0.290 |
| FXST | 0.020 | 0.016 | 0.202 | 0.003 | 0.015 | 0.837 |
| SLF | 0.077 | 0.017 | 8.1x10-6* | 0.017 | 0.010 | 0.076 |
| SFO | 0.067 | 0.016 | 2.0x10-5* | 0.022 | 0.011 | 0.054 |
| IFO | 0.046 | 0.018 | 0.010 | 0.005 | 0.015 | 0.756 |
| UNC | -0.005 | 0.020 | 0.768 | -0.016 | 0.019 | 0.414 |
| TAP | 0.014 | 0.017 | 0.429 | -0.039 | 0.012 | 0.0007* |

28 ROIs were analyzed in 3117 patients from 10 cohorts using linear mixed models. Study site was included as random effect and WMH volumes, age, sex and vascular risk compound score as fixed effects. WMH volumes were cube root transformed. WMH volumes and the vascular risk compound score were standardized (i.e. converted to z-scores). *Statistically significant after Bonferroni correction for multiple comparisons.

**Supplementary Table 3. Sensitivity analysis: association between regional WMH volumes and amyloid status in 1258 patients.**

| Region |  | Model 1: corrected for age, sex, study site | | |  | Model 2: additionally corrected for total WMH volume | | |
| --- | --- | --- | --- | --- | --- | --- | --- | --- |
|  | B main analysis (n=1273) | B sensitivity analysis (n=1258) | SE | p-value | B main analysis (n=1273) | B sensitivity analysis (n=1258) | SE | p-value |
| SCC | 0.281 | 0.281 | 0.052 | 7.4x10-8* | 0.103 | 0.106 | 0.029 | 0.0002* |
| PTR | 0.285 | 0.281 | 0.053 | 1.5x10-7* | 0.097 | 0.100 | 0.029 | 0.0004* |

In this sensitivity analysis, only patients with available data on both amyloid status and the VRCS were included. 28 ROIs were analyzed in 1258 patients from 6 cohorts using linear mixed models. Study site was included as random effect and WMH volumes, age, sex and vascular risk compound score as fixed effects. WMH volumes were cube root transformed. WMH volumes were standardized (i.e. converted to z-scores). *Statistically significant after Bonferroni correction for multiple comparisons.

**Supplementary Table 4. Sensitivity analysis: association between regional WMH volumes and vascular risk compound score in 1258 patients.**

| Region |  | Model 1: corrected for age, sex, study site | | |  | Model 2: additionally corrected for total WMH volume | | |
| --- | --- | --- | --- | --- | --- | --- | --- | --- |
|  | B main analysis  (n= 3117) | B sensitivity analysis (n=1258) | SE | p-value | B main analysis (n= 3117) | B sensitivity analysis (n=1258) | SE | p-value |
| MCP | 0.082 | 0.054 | 0.031 | 0.084 | 0.067 | 0.044 | 0.031 | 0.173 |
| GCC | 0.074 | 0.081 | 0.028 | 0.005 |  |  |  |  |
| BCC | 0.076 | 0.083 | 0.028 | 0.004 |  |  |  |  |
| CST | 0.062 | 0.091 | 0.031 | 0.003 |  |  |  |  |
| ALIC | 0.066 | 0.082 | 0.027 | 0.003 |  |  |  |  |
| ACR | 0.094 | 0.098 | 0.028 | 0.0005* | 0.034 | 0.029 | 0.016 | 0.068 |
| SCR | 0.101 | 0.125 | 0.028 | 7.6x10-6* | 0.038 | 0.055 | 0.014 | 0.0002* |
| PCR | 0.055 | 0.071 | 0.028 | 0.012 |  |  |  |  |
| EC | 0.103 | 0.073 | 0.028 | 0.009 | 0.052 | 0.022 | 0.022 | 0.331 |
| SLF | 0.077 | 0.048 | 0.026 | 0.063 |  |  |  |  |
| SFO | 0.067 | 0.084 | 0.028 | 0.002* |  |  |  |  |

In this sensitivity analysis, only patients with available data on both amyloid status and the VRCS were included. 28 ROIs were analyzed in 1258 patients from 6 cohorts using linear mixed models. Study site was included as random effect and WMH volumes, age, sex and vascular risk compound score as fixed effects. WMH volumes were cube root transformed. WMH volumes and the vascular risk compound score were standardized (i.e. converted to z-scores). *Statistically significant after Bonferroni correction for multiple comparisons.
